# Oscillations of ultra-weak photon emission from cancer and non-cancer cells stressed by culture medium change and TNF-*α*

**DOI:** 10.1038/s41598-017-10949-z

**Published:** 2017-09-12

**Authors:** Pierre Madl, Thomas Verwanger, Mark Geppert, Felix Scholkmann

**Affiliations:** 1 Department of Molecular Biology, University of Salzburg, A-5020 Salzburg, Austria; 2 Biomedical Optics Research Laboratory, Department of Neonatology, University Hospital Zurich, CH-8091 Zurich, Switzerland; 3 Research Office for Complex Physical and Biological Systems, CH-8038 Zurich, Switzerland; 4 ER-System, A-5440 Golling, Austria

## Abstract

Cells spontaneously emit photons in the UV to visible/near-infrared range (ultra-weak photon emission, UPE). Perturbations of the cells’ state cause changes in UPE (evoked UPE). The aim of the present study was to analyze the evoked UPE dynamics of cells caused by two types of cell perturbations (stressors): (i) a cell culture medium change, and (ii) application of the pro-inflammatory cytokine tumor necrosis factor alpha (TNF-*α*). Four types of human cell lines were used (squamous cell carcinoma cells, A431; adenocarcinomic alveolar basal epithelial cells, A549; p53-deficient keratinocytes, HaCaT, and cervical cancer cells, HeLa). In addition to the medium change, TNF-*α* was applied at different concentrations (5, 10, 20, and 40 ng/mL) and UPE measurements were performed after incubation times of 0, 30, 60, 90 min, 2, 5, 12, 24, 48 h. It was observed that (i) the change of cell culture medium (without added TNF-*α*) induces a cell type-specific transient increase in UPE with the largest UPE increase observed in A549 cells, (ii) the addition of TNF-*α* induces a cell type-specific and dose-dependent change in UPE, and (iii) stressed cell cultures in general exhibit oscillatory UPE changes.

## Introduction

Cells and tissues spontaneously emit photons in the UV to visible/near-infrared spectral range (approx. 200–1300 nm) even without photoexcitation (for a review see^1–4^). This spontaneous ultra-weak photon emission (UPE) with an intensity in the order of 10^1^–10^4^ photons/s cm^2^ ^3^ is not thermal radiation^1^, but considered to be mainly due to the de-excitation of energetically excited species (atoms, molecules) to a deeper energy level that is accompanied by the emission of photons. The energetic state of a molecule of atom (*E*_total_) is, according to the Born-Oppenheimer approximation, given as *E*_total_ = *E*_electronic_ + *E*_vibrational_ + *E*_rotational_ + *E*_nuclear_, with *E*_electronic_ the electronic energies (i.e., kinetic energies, electron-nuclear attraction as well as interelectronic and internulear repulsive forces), *E*_vibrational_ the vibrational energies, and *E*_nuclear_ the nuclear spin energy. Energy transitions of atoms or molecules are quantized involving the resonant absorption of incident energy and the subsequent quantized emission of it. In case of UPE, the excited energy levels involved refer to transitions regarding changes in *E*_vibrational_ and *E*_rotational_, associated with luminescence in the UV-IR optical spectrum. According to the conventional view, the energetically excited species are formed by oxidation reactions caused by radical or non-radical reactive oxygen (ROS) and nitrogen (RNS) species with lipids, proteins and nucleic acids^1, 5^. Especially the UPE in the visible/near-infrared region can be attributed to lipid peroxidation, protein and nucleic acid oxidation. As summarized nicely by Pospíšil *et al*.^5^ the chain reactions leading to UPE can be initiated by the oxidation of biomolecules that yield peroxyl and alkoxyl radicals. These radicals then recombine/cyclize to form dioxetanes or tetraoxides, which decompose to form triplets, excited carbonyl or singlet oxygen. The triplet excited carbonyls can emit photons or transfer energy to chromophores, or decay through another pathway. Three aspects in the generation of UPE-emission can be regarded: (i) oxidation of biomolecules (i.e., cycloaddition of singlet oxygen (^1^O_2_) and hydrogen abstraction by HO^•^), (ii) self-recombination of organic radicals (i.e., cyclization and self- recombination of peroxyl radicals (ROO^•^), forming high-energy intermediates, i.e. dioxetane (ROOR) and tetroxide (ROOOOR)) and alkoxyl radicals (RO^•^), and (iii) excited energy transfer to chromophores (e.g., flavins, melanin, chlorophyll, bilirubin, poryphyrins, urocanic acid). For a visualization of the processes involving UPE please see Fig. 1 in Pospíšil *et al*.^5^. Besides these mechanisms, other processes, such as proton flows through cytochrome oxidase enzymes in the mitochondrial membrane^6^ and transitions of excitons in proteins^7^, could also play a role for UPE generation. Particularly the former – although to our knowledge no mechanistic evidence has been published yet – yields photon emissions in the near IR-range at about 850 nm, while the latter presents theoretical results referring to photon emission in the longer wavelength range. Whereas UPE in the visible/near-infrared region can be attributed to the described mechanisms quite well, the origin of UPE in the UV region (especially < 350 nm) is not completely understood yet.

**Figure 1:**
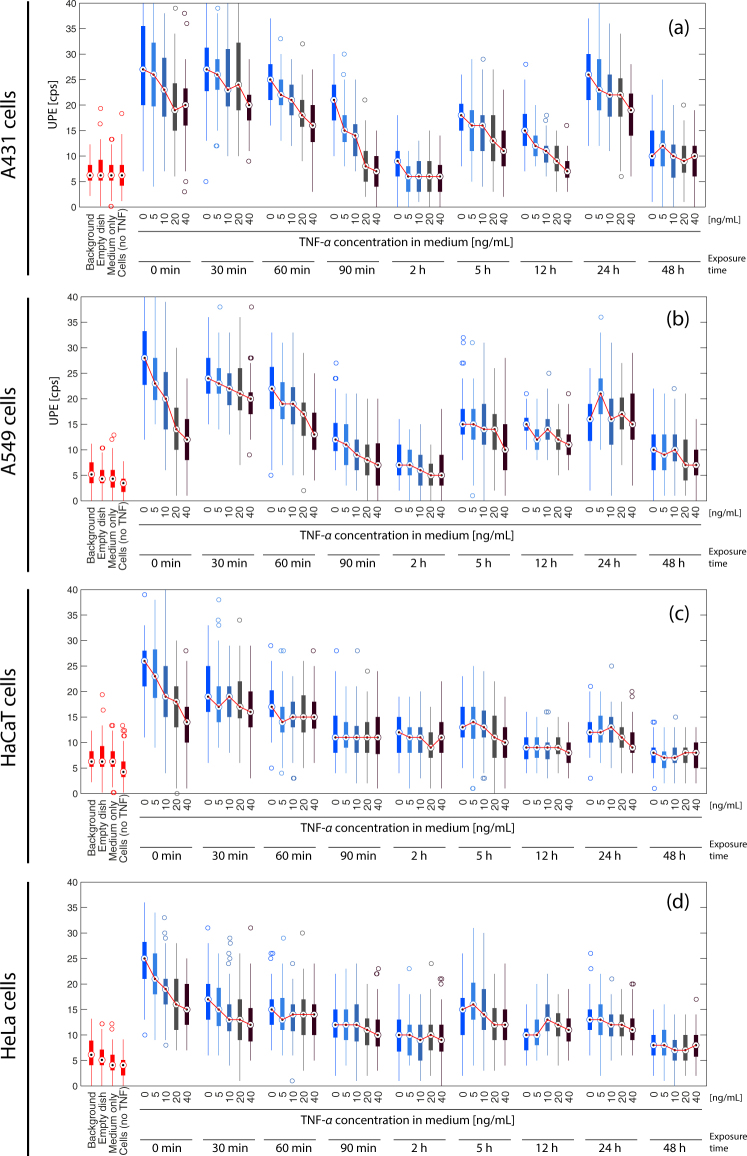
Time-series of UPE measurements of the four cell-lines used. The four initial readings in each graph relate to the background measurement batch (including cells not exposed to TNF-*α*). Subsequent readings relate to cells exposed to fresh medium (fresh medium change was made only at interval “0 min” along with the corresponding TNF-*α* concentrations).

ROS and RNS are constantly formed in cells, e.g., by mitochondria in the respiratory chain where superoxide (O_2_^•−^) is produced in the mitochondrial matrix (predominantly on complex I) which reacts to form H_2_O_2_ in subsequent steps^8^. The mitochondria-mediated ROS production rate accounts for 1–2% of the oxygen consumed *in vitro*, but the *in vivo* rate is far lower (approx. 0.1–0.4%)^8^.

The importance of oxidation reactions for the generation of UPE has been shown by a number of studies so far, demonstrating that (i) the addition of ROS or the stimulation of ROS production enhances the UPE intensity^9, 10^, and (ii) the application of ROS scavengers (antioxidants) decreases UPE^9–11^. The application of antioxidants can result in complex non-linear dose-response functions (e.g., glutathione in low doses (1 µM) increased UPE whereas in higher doses (500 µM, 1000 µM) it suppressed UPE, as recently demonstrated in differentiated human promyelocytic leukemia HL60 cells^12^). Such non-linear responses of UPE to external perturbations were also seen in experiments at elevated temperature^13^, upon electrostatic field exposures^14^, or upon incubation with aqueous solutions of ethidium bromide (*i.e*., increased UPE with low concentration, decrease with high concentration)^15^. Thus, the generation mechanisms of UPE might be an intrinsic phenomenon of cells and tissues as already pointed out in the 1990s by Gu and Popp^16^. Non-linearity is a very frequently observed property of biological systems^17^ but is also observed in non-biological systems^18^ so that its presence is not a direct proof for higher-order complex, i.e. biological, processes.

The qualitative characteristics of UPE (intensity and spectral composition) depend on the state and conditions of the biological system that emits UPE. Spontaneous UPE is correlated with the state of the cells and the conditions of the cellular environment, e.g., the cell cycle stage and proliferation activity^19–24^, temperature^22, 23, 25–27^, oxygen concentration^9, 24, 25, 28^, tissue blood flow^29^, or malignancy (tumor tissue exhibits higher UPE than normal healthy tissue)^30–33^.

What is evident from the experimental results obtained over the last decades is that the UPE characteristics are a unique marker for the state of the investigated biological system. In order to investigate the state of the system via UPE, two approaches can be generally applied: (i) measuring the spontaneous (i.e., non-evoked) UPE changes, or (ii) measuring the UPE changes evoked by a stimulus. Whereas *non-evoked* UPE changes already show a great variability and complexity (i.e., due to cell cycle-dependent activity, chronobiological state changes or also due to external influences like for example the luni-solar gravitational tide^34–36^), the *evoked* UPE changes exhibit an even increased complexity. Such changes are mainly due to (i) a stimulus applied to a biosystem, which results in triggering a great variety of different cellular processes in this system (which interact with each other resulting in emergent behaviour), and (ii) the specific stimulus-characteristics will translate non-linearly to the reaction of the system. Both aspects are the results of dynamic systems being in a thermodynamically non-equilibrium state with non-linear regulatory control^37, 38^. Based on these facts, experimental work involving stimulus-evoked changes in UPE needs to be interpreted carefully in order to draw correct conclusions about the causal relationship between the stimulus, or the stimuli, and the accompanied UPE change.

Garcia-Montero *et al*.^39^ demonstrated that changes of the cellular medium during experiments can elicit significant perturbations to the cell culture, i.e., “the renewal of culture medium induces a transient cellular stress that may be a source of artefacts in experiments performed shortly after a change of culture medium.” More specific, they detected that the change of medium in NIH 3T3 mouse embryonic fibroblast cells triggered a stress response involving the activation of p8 (an injury-activated gene), the mitogen-activated protein kinases p38, the c-Jun N-terminal kinases (JNKs), the stress-induced extracellular-signal-regulated kinases (ERK1/2) and the C/EBP*β* transcriptional factor. The up-regulation of the activity of p8, p38, JNK, ERK1/2 and C/EBP*β* is of significance since many genes are regulated according to the expression levels of these signalling molecules. The biological effect of a medium change was also observed in Swiss 3T3 cells where it was found that “changing the culture medium prior to stimulation resulted in an augmentation of bradykinin-induced prostaglandin E2 synthesis”^40^. These findings about the effects of a medium change have relevance to all UPE studies involving the measurement of cell cultures.

Recently, Doll *et al*.^41^ showed in cells of the mouse hippocampal neuronal cell line HT-22 and mouse primary cortical neurons that the pro-inflammatory cytokine tumor necrosis factor alpha (TNF-*α*) caused mitochondrial dysfunction. Interestingly, the effect of TNF-*α* on the oxygen consumption rate of the cell culture exhibited a non-linear (oscillatory) dynamical behaviour indicating the activation of a compensatory mechanism to respond to the mitochondrion-toxic TNF-*α* exposure. However, this response was found to be cell type-dependent and evident only at a long observation period (up to 12 hours).

The findings of Garvia-Montero *et al*.^39^ and Nakatani *et al*.^40^ concerning the stress-induction due to medium change, and the work of Doll *et al*.^41^ (concerning the complex effects of TNF-*α* on mitochondrial functions), motivated us to investigate: (i) the effect of a medium change on the UPE of cell cultures, and (ii) the effect of TNF-*α* (of varying concentration and with different exposure times) on the UPE dynamics of cell cultures. To this end, four different cell types were analyzed using a newly developed high-sensitive photomultiplier system. Such an investigation has not been performed yet. The result of the investigation is of relevance not only for basic research involving the measurement of UPE from cell cultures but also because the impact TNF-*α* on mitochondrial function is increasingly recognized since it has a relevance for medical research due to the involvement of TNF-*α* in many pathophysiological processes^42–47^.

## Materials and Methods

### Cell cultures

Four types of cell lines were used for the experiment of which the first three were obtained from the Leibnitz Institute DSMZ-German Collection of Microorganisms and Cell Cultures: (i) human squamous cell carcinoma cells (A431, ATCC-No. CRL-1555), (ii) adenocarcinomic human alveolar basal epithelial cells (A549, ATCC-No. ACC - 107), (iii) cervical cancer cells (HeLa, ATCC-No. ACC - 57), and (iv) human p53-deficient keratinocytes (HaCat, ATCC-No. ACC-771) – with the latter being kindly provided by the DKFZ Heidelberg, Germany.

Handling of cell lines and addition of media was performed under a HEPA-ventilated laminar flow hood, whereas incubation of cells took place at 97% rH, 5% pCO_2_, and at 37 °C in an Heraeus Cytoperm 8080 incubator (Thermo Scientific, Braunschweig, Germany). Upon reaching confluency, cultures have been used straight away. In few exceptional cases, the medium was changed every other day, and cells were sub-cultured 1:4 until 80–90% confluency was reached.

### Chemicals and reagents

#### Media and supplements

All ingredients were obtained from Sigma-Aldrich (Schnelldorf, Germany).

A431 cells were cultivated in Dulbecco’s modified Eagle’s medium (DMEM) supplemented with 5% fetal bovine serum (FBS), 2 mM L-glutamine, 1 mM Na-pyruvate, 100 U/mL penicillin and 0.1 mg/mL streptomycin.

HaCaT cells were cultivated in DMEM supplemented with 10% FBS, 2 mM L-glutamine, 1 mM Na-pyruvate, 100 U/mL penicillin and 0.1 mg/mL streptomycin.

A549 cells were cultured in RPMI Medium supplemented with 10% FBS, 2 mM L-glutamine, 100 U/mL penicillin and 0.1 mg/mL streptomycin.

HeLa cells were cultured in DMEM supplemented with 10% FBS, 2 mM L-glutamine, 100 U/mL penicillin and 0.1 mg/mL streptomycin.

#### TNF-α enriched media

DMEM (described above but without FBS) added to each; 40, 20, 10, 5 ng/mL stock solution.

#### Seeding

For experimental incubation, cells were seeded in 60 mm polystyrene (PS) petri dishes (Sarstedt, Newton, USA) at a density of 10^5^ cells/cm^2^ in 4 mL culture medium and incubated for 24 h to reach approximately 90% confluency before treatment with TNF-*α* commenced according to section 2.4.

### Ultra-weak photon emission measurement setup

In order to record spontaneous UPE, the detector consists of a head-on, low-noise 95xx-series Photo-Multiplier Tube (PMT) with a nominal aperture of 51 mm provided by Electron Tubes (Uxbridge, UK). In order to increase the signal-to-noise-ratio (i.e., minimizing the thermal noise of photon-converting cathode), the PMT used is embedded in a Peltier-based cooling unit (Fact-50 housing, from the same supplier). Since condensation of water-vapour at the photon-converting layer must be inhibited, the Dewar-like arrangement is fitted with a double-layered quartz-glass separator window. The evacuated cavity in-between these layers further improves the insulating properties, while Quartz-glass itself allows photons in the UV-range to reach the photon-converting cathode. Signal amplification, discrimination and TTL-converter of the PMT-output likewise came from that supplier. Dark-room conditions are obtained by placing these components along with an electro-mechanical shutter (Melles Griot, Rochester, USA) IES-series with 64 mm aperture and controller into an aluminium, light-tight casing with a removable lid to gain access to the measurement compartment. Data acquisition and processing was achieved by an extended LabView^®^ solution; i.e. the suppliers core code has been embedded into a specifically for this detector system developed user-interface enabling extended operational procedures in order to increase multi-purpose applicability of the UPE-detector system. An in-depth description of the detection system can be found as supplementary material.

### Experimental protocol: medium change and TNF-α treatment

Around 90% confluence in all of the four cell lines was achieved about 24 h after inoculation onto the 60 mm PS dishes. In none of the four cell lines did coverage reach 100%, yet they self-stabilized at a critical, cell-specific density and remained constant for the entire series of measurement (typically 24 h). To exclude material-induced artefacts, UPE-measurements of both media- and PS dishes alone were done. Only with this approach it was feasible to determine cell-specific UPE-intensities using media-covered cells in PS dishes that lacked TNF-*α*. Media change at time “0” was performed under a sterilized and HEPA-ventilated hood whereby all dishes underwent exchange with fresh medium along with a gradually increasing concentration of TNF-*α* (0, 5, 10, 20, 40 ng/mL).

Trials with cultures exposed to the various TNF-*α* concentrations but without undergoing medium change have not been performed. The decision not to do so derived from a preliminary trial executed prior to this experimental series in which it was observed that cells experiencing medium change require a latency period up to 12 h until the reverberating effects in UPE faded out significantly. After this latency period a discrimination of the introduced TNF-*α* stimulus becomes more reliable. However, working with cultures that experienced an earlier medium change would introduce other effects that render the gathered UPE-data for the time slot between 24 to 48 h subject to additional artefacts (accumulation of metabolites, microbial interference, etc.).

By operating the UPE-system in spontaneous emission mode and culture handling under dimmed light conditions the cell-samples one by one were taken from the incubator and placed into the dark-chamber. After an initial acclimatisation time-slot whereby cells approached a steady-state emission rate (typically after around 10–20 s) the actual measurement was initiated. Due to the sheer number of plates to measure (5 for each cell-line) each measurement cycle was limited to 60 s – only the controls have been done for 180 s. Regardless of the duration, the hardware setup operated with a sampling rate of 1 per second.

Our protocol was set to a sampling frequency of 0, 30 60, 90, 120 min with subsequent increases in sampling intervals to 2, 5, 12, 24 and 48 h after initial medium change. In-between measurements and in order to minimize abiotic stress to the cells, the plates were stored in the incubator. A tight sampling rate like this allows screening of one cell line per day only, or a full week for all four cell-lines.

### Data analysis and statistics

Statistical analysis was performed using Matlab (R2013b, MathWorks, Natick, Massachusetts, USA). The Wilcoxon rank sum test was used to compare groups of samples. Values are expressed as median ± median absolute deviation (MAD). Differences were considered significant at *p* < 0.05.

The effect size was quantified by calculating Cohen’s *d* in a robust version (concerning outliers) according to *d* =  *x*_2,med_ − *x*_1,med_/*s*, with *x*_1,med_ the median of sample *x*_1_, *x*_2,med_ the median of sample *x*_2_, and *s* the pooled median absolute deviation for the two samples.

## Results

### Change of culture medium (without added TNF-*α*) induces a cell-type specific transient increase in UPE

**Figure 2:**
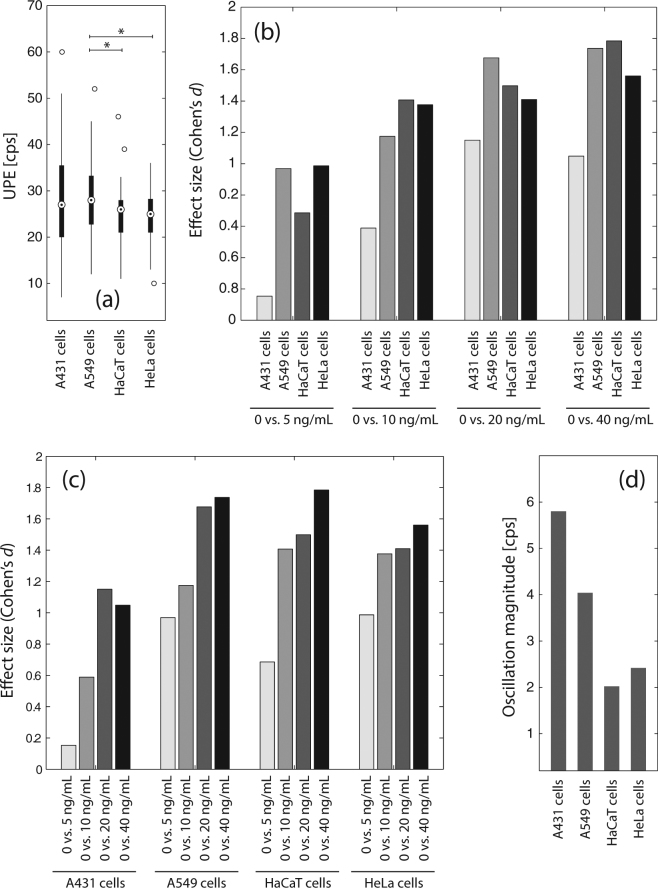
(**a**) Change of culture medium (without added TNF-α) induces a cell-type specific transient increase in UPE. (**b**,**c**) UPE-reducing effect due to the presence of a given TNF-*α* concentration. The strength of reducing the medium change induced UPE is largest in HaCaT cells (@ 40 ng/mL TNF-*α*) and smallest in A431 cells (@ 5 ng/mL TNF-*α*). (**d**) UPE oscillation magnitude depending on the cell type.

In all four cell types the change of the culture medium induced a large increase in UPE (A431 (median ± MAD): 27 ± 7 counts per second (cps), A549: 28 ± 5 cps, HaCaT: 26 ± 3 cps, HeLa: 25 ± 4 cps) (see Figs. 1 and 2(a)). The UPE increase in A549 cells was not only statistically significant (*p* < 0.05) but also larger compared to the UPE increase in HeLa and HaCaT cells.

### Change of culture medium in combination with TNF-*α* addition induce cell-type specific and dose-dependent changes in UPE

As shown in Fig. 1, cells in a medium containing TNF-*α* exhibited an increase in UPE. However, the TNF-*α* induced UPE increase was less than the increase caused by the medium change alone. The TNF-*α* addition significantly (*p* < 0.05) reduced the evoked UPE increase due to the medium change in all cell types.

The larger the TNF-*α* concentration added, the larger the UPE reducing effect size (quantified by Cohen’s *d*) was (correlation strength: *r* = 0.7163, *p* < 0.01; averaged over all cell types) (see Fig. 2(b)).

As visualized in Fig. 2(c), the effect size (i.e., the strength of reducing the medium change induced UPE increase due to TNF-*α* addition) was dependent on the cell type, with the largest effect size (*d*) of 1.7844 in HaCaT cells (when treated with 40 ng/mL TNF-*α*) and the smallest of 0.1536 for the A431 cells (when treated with 5 ng/mL TNF-*α*).

### Stressed cell cultures (due to medium change with and without TNF-*α* added) exhibit oscillatory UPE changes

**Figure 3:**
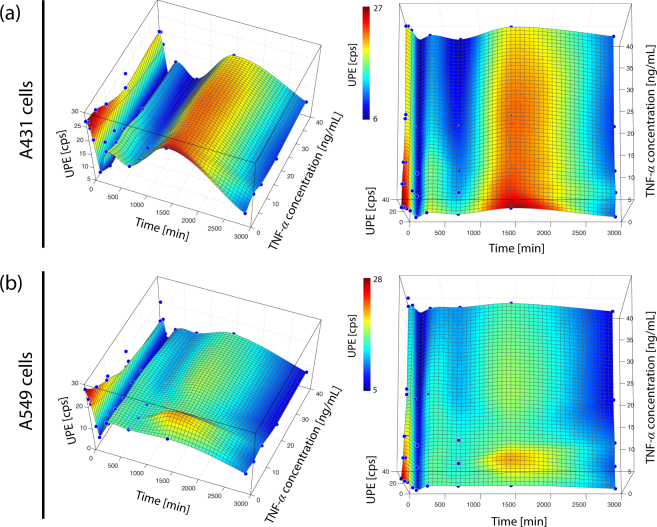
Non-monotonic time-dependent UPE behaviour of (**a**) A431 and (**b**) A549 cells. The magnitude of the oscillation reflects both stimulus and cell type dependency.

**Figure 4:**
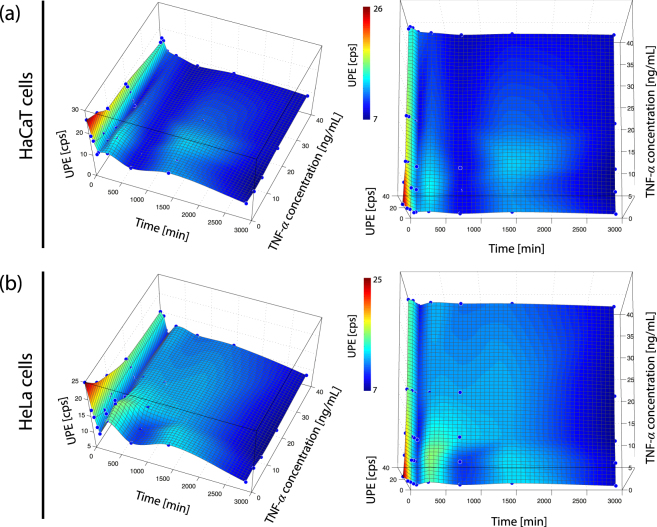
Non-monotonic time-dependent UPE behaviour of (**a**) HaCaT and (**b**) HeLa cells. The magnitude of the oscillation reflects both stimulus and cell type dependency.

A non-monotonic (oscillatory) time-dependent UPE change was observed in all four cell types and for both, the cell stimulation due to the medium change as well as due to the TNF-*α* incubation. The time-dependent dose-response consists of five phases: (i) a gradual decrease of UPE (phase 1, incubation time span: 0–2 h), (ii) an UPE increase with respect to the end of phase 1 (phase 2, incubation time: 5 h), (iii) an UPE decrease with respect to phase 2 (phase 3, incubation time: 12 h), (iv) an UPE increase with respect to phase 3 (phase 4, incubation time: 24 h), and (v) an UPE decrease with respect to phase 4 (phase 5, incubation time: 48 h) (see Figs 1, 3 and 4 (visualized with a equidistantly spaced time-axis and in 2D). The oscillation can thus be described by a damped oscillation having two peaks (at 5 h (phase 2) and 24 h (phase 4)).

The magnitude of the oscillation depended of the type of stimulus as well as the cell line: (i) the medium change induced the strongest oscillation but it was also visible for the TNF-*α* treated cells, and (ii) the oscillation magnitude (quantified as the standard deviation of the mean of UPE values [due to stimulation via medium change and TNF-*α*] for periods 1–5) showed a cell-type dependency (see Fig. 2(c)). A431 cells exhibited the strongest oscillation (5.7958 cps), HaCaT the weakest (2.0822 cps). The oscillation magnitude of the A549 cells was similar to that of the A431 cells (i.e., 4.0495), whereas HeLa cells showed a similar value as the HaCaT cells (i.e., 2.4182).

## Discussion

### UPE increase due to culture medium change: what is the cause and why is it cell-type dependent?

Our observation that a cell medium change is associated with an UPE increase is in generally agreement with findings of Nakatani *et al*.^40^ and Garcia-Montero *et al*.^39^. The former authors observed an augmentation of bradykinin-induced prostaglandin-E2 synthesis in Swiss-3T3 fibroblasts upon change of culture medium. Under normal culture conditions these cells produce only a small amount of prostaglandin-E in response to bradykinin. They have shown that the augmented prostaglandin-E synthesis was not only attributable to medium change but also to the duration of exposure of the cells to the fresh medium – the response window was found to last from 30 min to 4 h with a peak response following 60 min after the performed swap with fresh medium.

Similarly, Garcia-Montero *et al*.^39^ demonstrated that a medium change elicited significant perturbations to cell cultures (NIH 3T3, HeLa, IEC-6, TC7, HT29, AR4-2J, LS174T, SW480), resulting in the transiently increased expression of p38 (peak at 60 min), JNK (peak at 15–30 min), ERK1/2 (peak at 15 min), C/EBP*ß* (peak at 2–3 h), and p8 (peak: 4–6 h). However, in our experiment we observed an immediate increase in UPE (at *t* = 0 min), which decayed quite fast, i.e., a reduction was observed already at the second measurement (at *t* = 30 min), we presume that the observed UPE increase was not directly linked to a possible increased stress-induced gene expression of p38, JNK, ERK1/2, C/EBP*ß* or p8.

Assuming that the ROS produced by mitochondria^8^ are the main source of chemical reactions leading to UPE, we hypothesize that the culture medium change induces a transient change in mitochondrial function. Mitochondrial ROS production is mainly determined by the mitochondrial membrane potential (ΔΨ_m_) and the redox environment (RE). Changes in either ΔΨ_m_ or RE are associated with ROS levels, i.e., ROS levels have a maximum when mitochondrial respiration is maximal and the RE is in an intermediate state between a low-energy oxidative phase and a high-energy reductive phase^48^. Cancer cells have an already higher ΔΨ_m_ value compared to mitochondria in healthy cells (approx. −220 mV vs. −140 mV)^49^. The increased UPE due to the medium change could be thus principally explained by either a further increase in ΔΨ_m_ or a change in the RE, most probably a shift to a more oxidative state.

In our study, the UPE induced by the medium changes was dependent on the cell type: (i) the strongest UPE increase was observed in A549 cells, and (ii) the UPE increase in A549 and A431 was stronger than in HaCaT and HeLa cells. Interestingly, when comparing the cells with respect to their invasiveness, the same cell type-dependent pattern is evident (invasion score for A431: 0 ± 0, for A549: 0 ± 0, for HaCaT: 0.98 ± 0.18, and for HeLa: 1.01 ± 0.10)^50^. But is there a link between the invasiveness of cancer cells and the magnitude of UPE increase due to a medium change? The degree of invasiveness is related to the mitochondrial redox signalling, *i.e*., invasive cancer is associated with an increased production of ROS by mitochondria which increases invasiveness (*i.e.*, H_2_O_2_ stabilizes the hypoxia-inducible factor 1-*α* (HIF-*α*), leading to an increased expression of pro-tumor cytokines driving the cell to a more invasive tumor cell phenotype)^51^. Thus, the cell type-specific increase of UPE due to the medium change may be to the different susceptibility of the cancer cells to external stressors: whereas A431 and A549 cells (low invasiveness) are more susceptible, HaCaT and HeLa seem to be more robust – possibly due to the different cancer cell phenotypes (e.g., degree of glycolytic shift).

### UPE increase due to culture medium change and TNF-*α* addition: what is the cause and why is it cell-type dependent?

Since TNF-*α* causes mitochondrial dysfunction accompanied by an increased production of ROS (mitochondrial oxidative stress)^41, 52–54^ (e.g., peroxinitrite (ONOO^−^)^55, 56^, O_2_^•−^^57^, and increased concentration of oxidized glutathione disulfide (GSSG)^56^), and since oxidative stress is associated with increased UPE^2, 58^, it was expected that the addition of high doses of TNF-*α* to the cell cultures would cause a significant increase in UPE. However, the measured data show that TNF-*α* had the opposite effect, *i.e*., seemingly down-regulating the medium change-induced stress-related UPE response. Furthermore, the higher the concentration of applied TNF-*α*, the weaker was the UPE increase. This represented a finding which was not expected due to the simple assumption that any substance that boosts ROS production would be accompanied by an UPE increase.

Our results indicate that the effect of simultaneously applying two stressors (medium change and TNF-*α* exposure) caused a non-additive reaction of the cells, suggesting that either both stressors interact with each other so that the overall stress signal was diminished, or that the cell’s reaction is different when both stressors are applied simultaneously. One possibility would be that the combined application of the two stressors induced such a strong cellular stress that the cells significantly increased the self-protection mechanism, causing a reduction of the overall oxidative stress, and thus UPE.

The variable susceptibility of the investigated cells to the combination of stressors may be due to the different densities of receptors on the cell surface such as the epidermal growth factor (EGF) receptor (EGFR). EGFR is present on all the four investigated cell types^59, 60^, but with different densities (A431: 636 molecules/µm^2^, HeLa: 270 molecules/µm^2^, A549: 142 molecules/µm^2^)^60^. A431 cells have thus a significantly higher EGFR density compared to the other cell lines, which lead us to ask whether EGFR density could be linked to the observation in our experiment that A431 cells showed overall the smallest effect size (both for the medium change and for the combined stressors, i.e. medium change and TNF-*α* exposure). TNF-*α* activates EGFR and increases the expression of EGFR^61, 62^. Activation of EGFR is in turn followed by a stimulation of the cells’ defence mechanisms against toxins (*i.e*., increased expression of detoxification enzymes and drug efflux pump proteins) via the EGFR-P13K-Akt/ERK MAPK signalling pathway^63^. Another cell defence mechanism, especially against TNF-*α*, is the ability of cells to release TNF-*α*-binding proteins (TNF-BPI, TNF-BPII), which have a TNF-*α* scavenging effect. The concentration of released TNF-*α*-binding proteins differs from cell to cell, e.g., HaCaT and A431 cells (HaCaT, TNF-BPI: 4008 ± 422 pg/mL, TNF-BPII: 301 ± 33 pg/mL ; A431, TNF-BPI: 3012 ± 305 pg/mL, TNF-BPII: 322 ± 35 pg/mL)^64^ – a fact maybe linked to our observation that the strength of reducing the medium change induced UPE increase due to TNF-*α* addition was dependent on the cell type, with the largest effect in HaCaT cells (when treated with 40 ng/mL TNF-*α*) and the smallest in A431 cells (when treated with 5 ng/mL TNF-*α*).

### *UPE oscillations in stressed cell cultures (due to medium change with and without TNF-α added): what is the cause and* why *is it cell-type dependent?*

One of the interesting findings of the present study was the observation of a non-monotonic (oscillatory) time-dependent UPE change in all four cell types and for both stimulations, i.e., the medium change as well as the medium change combined with TNF-*α* incubation. There are several possible mechanisms that could be responsible for this dynamics. These include cell-density changes due to proliferation, circadian rhythm of cellular processes, non-linear dynamics of mitochondrial function (ROS and cytochrome-*c* release, as well as oxygen consumption), or time-dependent up-regulation of adaptive stress responses. In the following subsections we discuss these possible factors in detail.

### Cell-density changes due to proliferation

During the time-course of the experiment (0–48 h) obviously cell proliferation took place. There are reports showing that proliferative activity can exhibit non-linear changes, e.g., aperiodic oscillations following the laws of deterministic chaos^65^. That the UPE from proliferating cells can exhibit non-stationary dynamics was shown by Galle *et al*. who demonstrated that the UPE intensity of the planktonic crustacean *Daphnia magna* was density-dependent in a non-linear way with 2–3 maxima occurring when the density of animals was increased from 1 to ~250^66^. However, in cell cultures this behaviour has not been observed yet. It was rather detected that an increased density of cells due to proliferation is characterised by three phases: first a slight decrease in UPE over a few hours, then a fast decrease, and finally a saturation phase^67^. Taken together, the oscillation observed in our study seems to be obviously related to non-stationary proliferation dynamics.

In a study with HeLa cells Kim *et al*.^68^ reported a strong correlation between changes in UPE intensity and changes in the proliferative rate of viable cells. Measuring also the UPE intensity at 24 h and 48 h (as in our study), Kim *et al*. observed an increase in UPE at 48 h compared to 24 h; in our study we observed a decrease, however.

### Circadian rhythms of cellular processes

Another possibility is that intrinsic chronological oscillations in the cells could cause the UPE changes. In HeLa cells such a circadian oscillation has been reported to be absent^69^, whereas in HaCaT cells it has previously been described^70^, and has been observed upon stimulation by temperature^71^. Concerning circadian rhythms in A431 and A549 we are not aware of reports published yet.

Ultradian and circadian rhythms of the activity of NAD(P)H oxidase (NOX) proteins (*i.e*., ECTO-NOX proteins) at the external surface^72–74^ may be linked to the observations in the present study, however, the observed oscillations showed a frequency change not expected and documented for ECTO-NOX based oscillations.

In this study the observed UPE oscillation was present in all investigated cells (A431, A549, HaCaT and HeLa) and did not have a constant frequency. We therefore rule out the possibility that a circadian oscillation can explain our observation.

### Non-linear dynamics of mitochondrial function: ROS and cytochrome-*c* release

**Figure 5:**
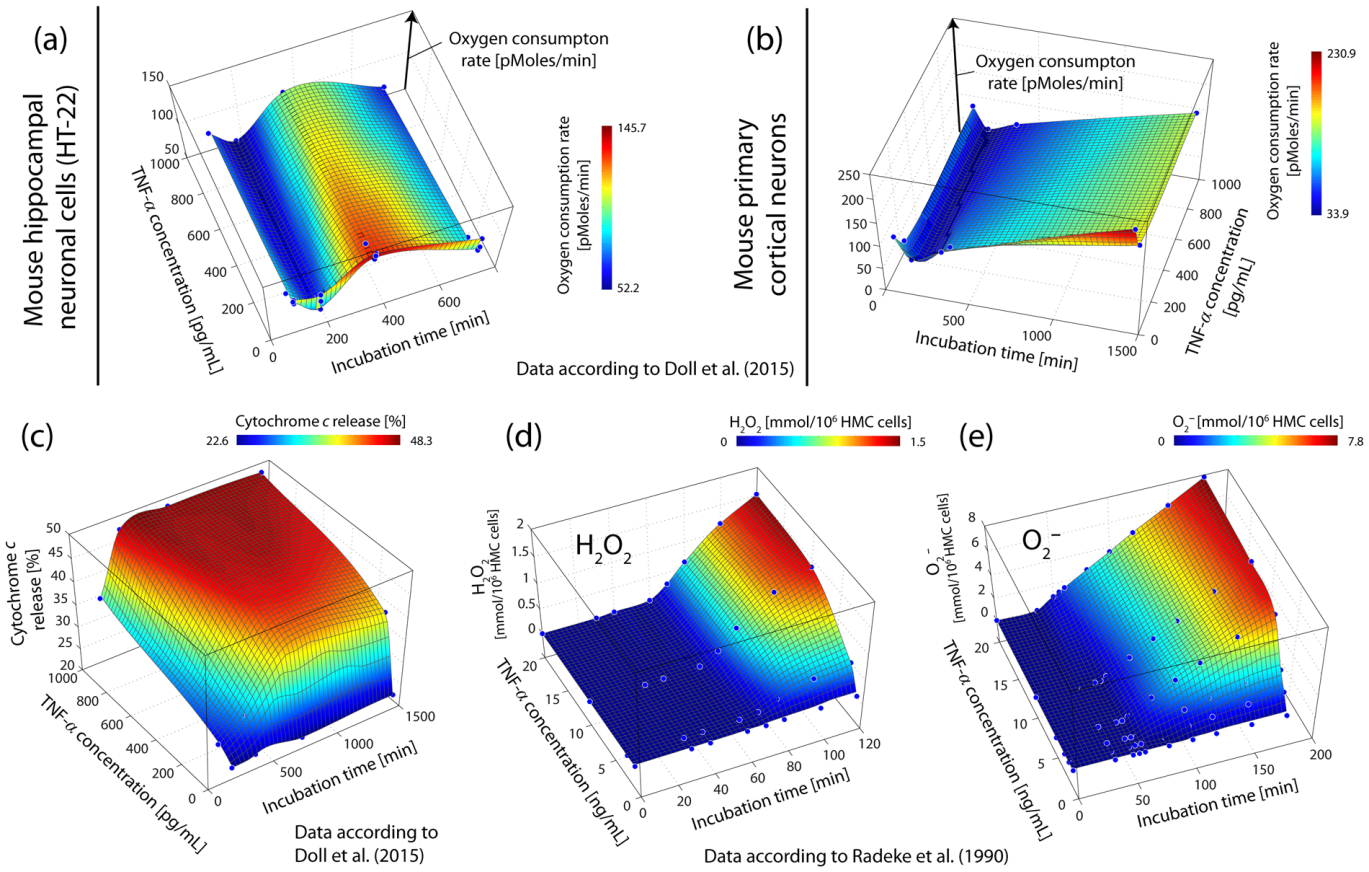
Effects of TNF-*α* on neurons from mice (**a**,**b**). A time-dependent oscillation of the oxygen consumption rate during the incubation HT-22 cells is visible in (**a**), whereas (**b**) depicts primary cortical neurons that do not reveal oscillatory behaviour. Similarly, does Cytochrome-*c* release of TNF-*α* incubated HT-22 cells of rodents not display an oscillatory pattern (**c**), indicating that oscillation might be related to ROS^41^. Likewise yield H_2_O_2_ and O_2_^•−^ the release of TNF-*α* in stimulated HMCs (**d**,**e**) a monotonic increase^75^.

Stress stimuli may cause non-stationary generation of ROS and/or cytochrome-c release from mitochondria is another theoretical possibility. However, it was shown that a TNF-*α* induced release of H_2_O_2_ and O_2_^•−^ follows a monotonic increase (following approximately a sigmoidal function)^75^ in human mesangial cells (see Fig. 5(d,e)). The experiment investigated only an incubation time of maximally 120 min though, so that it cannot be fully ruled out that the ROS dynamics show oscillations if the measurement would had been performed up to 48 h (as in our study). Doll *et al*.^41^ could demonstrate that cytochrome-*c* release of evoked by TNF-*α* incubation in the immortalized mouse hippocampal cells (HT-22) is characterized by a continuous increase until a saturation is reached (at approx. 12 h of TNF-*α* exposure) (see Fig. 5(c)). No oscillation was observed during this investigated incubation time (i.e., 3–24 h). Thus, we cannot rule out that the observed UPE oscillation is due to oscillations in ROS but an experimental confirmation regarding this possibility is lacking at the moment.

### Non-linear dynamics of mitochondrial function: oxygen consumption (metabolism)

A further possibility is that the mitochondrial metabolism, as indicated by the oxygen consumption, follows non-stationary dynamics in response to a stress stimulus. Indeed, an oscillation of the oxygen consumption rate during the incubation of HT-22 cells (total duration: 12 h) was observed with minima in the oxygen consumption at 3 h and 12 h and a peak at 6 h^41^ (see Fig. 5(a)). The oscillation was cell type-dependent (i.e., not present in mouse primary cortical neurons (see Fig. 5(b)), showed an increasing period length during the measurement, and was also present when the HT-22 cell culture was not exposed to TNF-*α* ^41^.

Whereas the latter two observations were also made in the UPE dynamics in our experiment (i.e., increased period length and presence also without TNF-*α*), an oscillation could be detected in our experiment in all four investigated cell lines. We therefore concluded that the changes in metabolic state of mitochondria may be responsible for the observed changes in UPE.

### Time-dependent upregulation of adaptive stress responses

Doll *et al*. hypothesized that the oscillatory dynamics they observed in mitochondrial respiration due to TNF-*α* exposure could be due to the fact that “cells appear to activate compensatory mechanisms to respond to the mito-toxic effects of TNF-*α*”^41^. The mechanism seems to be able to counterbalance the toxic effects for a specific period of time but later on is not able to compensate for the increasing cytotoxic effects that finally lead to significant cellular damage and even cell death.

In our opinion, such an adaptive stress response that consists of an active mito-toxic compensatory mechanism could be also responsible for the oscillatory UPE we observed in our experiment. This compensatory mechanism seems to involve changes in mitochondrial metabolism (explain the findings discussed in section 4.3.4) and possibly the upregulation of protective factors. For example, heat shock proteins (HSP) can be activated by TNF-*α* ^76, 77^, which lead to enhanced cell survival^78, 79^. Also glutathione (GSH) plays an important role in limiting TNF-*α* induced cytotoxic effects^80^. Especially the GSH transport in mitochondria seems to be crucial with regard to the defence against TNF-*α* induced oxidative stress^81^. Possible non-linear dynamics in HSP and GSH expression/activity may lead to the oscillations observed in UPE in our experiment.

## Conclusion

The findings of the present study link to previous observations by Nakatani *et al*.^40^ and Garcia-Montero *et al*.^39^ who showed cell stress-induction due to a medium change. Our findings furthermore corroborate the work of Doll *et al*.^41^ regarding the non-monotonic effects of TNF-*α* on mitochondrial functions. Taken together, these results are relevant for a basic understanding of UPE measurements in *in vitro* cell cultures. Furthermore, UPE measurements may have a potential for investigations with regard to the impact of TNF-*α* on mitochondrial function and its role in a number of pathophysiological processes^42–47^. Moreover, the stress-related responses were shown to be tied to the TNF-*α* concentration in which higher concentrations had a larger UPE reducing effect. Since we observed the UPE up to 48 h, this extensive observation window allowed us to identify a non-monotonic (oscillatory) time-dependent UPE change in all four cell types for both, medium change induced stimulation as well as TNF-*α* exposure in various concentrations. The damped oscillations are characterized by two peaks at 5 h (phase 2) and 24 h (phase 4).

Our findings revealed that a higher TNF-*α* concentration weakened the medium change induced UPE stress-signal responses – this was indeed a surprising observation. Obviously, overall oxidative stress reduction, mirrored in suppressed UPE, is tied to TNF-*α* concentrations. Altogether, the combined application of two stressors induced such a strong cellular loading that the cells responded by significantly increasing their self-protective mechanisms.

In alignment with the aforementioned studies^39, 40^ our data suggest that transient cellular perturbation generates artefacts in successive experiments performed shortly after medium change. In order to prevent stress-related artefacts of *in-vitro* cell studies, we follow the line of argument of Garcia-Montero *et al*. who suggested that rather than executing a full media swap at once to add 25% of conditioned medium to fresh medium^39^.

## Electronic supplementary material

Supplementary information
