# Supplementary material for: Oscillations of ultra-weak photon emission from cancer and non-cancer cells stressed by culture medium change and TNF-α
Source: Sci Rep. 2017 Sep 12;7:11249. doi: 10.1038/s41598-017-10949-z (PMC5596028; doi:10.1038/s41598-017-10949-z)
Supplement: Supplementary file 1 — Supplementary information [file 41598_2017_10949_MOESM1_ESM.doc]

Supplement Material for the manuscript for submission to *Scientific Reports*

# Oscillations of ultra-weak photon emission from cancer and non-cancer cells stressed by culture medium change and TNF-α

# Pierre Madl, Thomas Verwanger, Mark Geppert, Felix Scholkmann

The following core components of the *Coherence Monitor* are necessary to monitor ultra-weak photons in spontaneous emission mode (Fig. S1).

Core units and essential peripherals are available from ET-Enterprises (2016a) and consist of:

**Photo-Multiplier Tube (9558QB)**: This PMT consists of 52 mm diameter end window photomultiplier, with S20 infra-red sensitive photocathode, and 11 high-gain, high-stability, SbCs dynodes of the long-established venetian blind design providing a low after-pulse rate. The employed PMT-window characteristics cover a band from 160 – 870 nm with a corresponding refractive index of 1.46.

**PMT-signal amplification (AD-2)**: An amplifier-discriminator circuit combines a fast amplifier and a fixed-threshold discriminator with an overall sensitivity of approximately 1 mV. With the gain of the photomultiplier optimised, photon counting with a high signal to noise ratio and stability can be achieved up to count rates of 100 MHz. In addition, the unit is fitted with an adjustable input threshold (in the range from -0.5 to -2.5 mV), yielding an additional advantage where setting a higher threshold is necessary to eliminate pickup from an electrically noisy environment.

**ECL-TTL Converter (ET-1)**: In combination with the amplifier, this converter is designed for use in photon counting applications using a TTL digital counter. The unit employs fast logic circuits capable of driving TTL signals into a 50 Ω load. The output rests at a TTL high level (>2.4 V) and the leading edge of an input impulse will cause the output to fall to a TTL low level (<0.3 V) for the duration of the input pulse.

**Counter-Timer TTL-unit**: is a high performance pulse counting instrument for use with a PC or Laptop via the USB 2.0 interface that is also used to cover its power ratings. It can be operated as a rate-meter as a wide dynamic range photon counting system. A LabView® virtual instrument program option is included and was embedded into the system to design the *Coherence Monitor* (Madl et al. 2016).

**PMT HV-Unit (PM20D)**: This power supply provides a stable well regulated output controllable from 0.02 to 2 kV. It has been designed primarily for photomultipliers with the particular needs of the laboratory or research specialist in mind. The polarity is determined by selecting either the positive or negative output SHV socket mounted on the rear of the unit. The dual socket output guards against accidental polarity reversal. The unit offers additional safety features such as protection against overload, flashover and short circuit.

Additional peripherals to make cell-culture studies possible (ET-Enterprise, 2016b):

**PMT Cooling Unit & Power Supply (FACT50)**: To suppress dark-current formation and thereby increasing low level photon detection efficiency an air-cooled, 120 W thermoelectric cooling unit is used. The unit operates with Peltier Elements (PE), thus offering the advantage of no moving parts - except for the fans. The photocathode is set 31.8 mm behind the front surface of the flange and behind an evacuated, double- walled quartz-glass window with a lower cut-off window at 170 nm allowing the unit to remain condensation-free. In order to facilitate easy operation, the unit is supplied with a mains driven power supply, including a temperature control unit, digital temperature read-out and interconnecting cables.

**High-Speed Shutter (04IES215) & Controller (04ISC850)**: Shutter operation for the PMT utilizes a standard Melles-Griot (2016) shutter-controller unit with an aperture of 64 mm. The GVI solenoid drive, spring return shutters offer high performance in a range of operating conditions. The shutter is normally closed and operates at 12 V. The shutters are operated via the standard Melles-Griot 12 V IES controller, which is fitted with built-in control functions (including eight pre-set shutter speeds) as well as a remotely operated trigger port. Power to the shutter-controller unit is provided by a12 V unit (04IPS835) designed for use with IPS Electronic Shutter Controllers. The minimally required triggering time to activate the shutter should be >20 ms @ 12 V, whereas the holding voltage can drop to 6 V. The maximum duty cycles possible with these shutters are 45 ms (approx. 22.2 Hz); significantly higher rates may damage the solenoid as it may overheat.


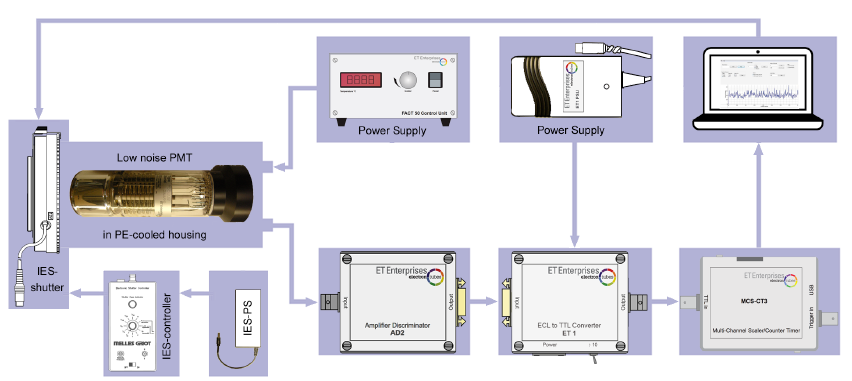


Fig. S1: Schematics of core unit of *Coherence Monitor* consisting of an ultra-weak Photon Counting System.


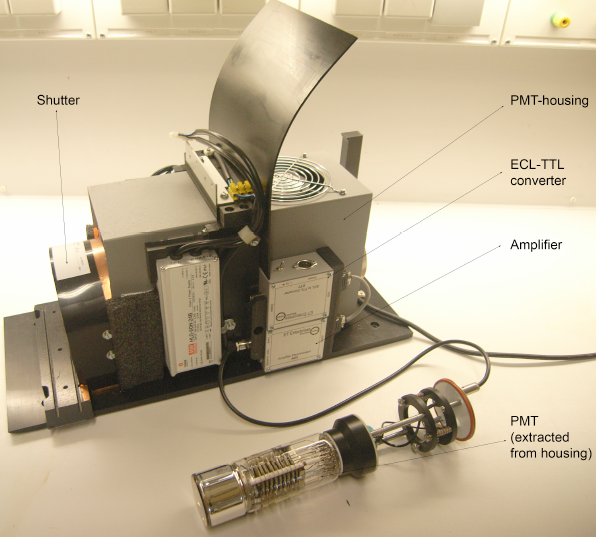


Fig. S2: Actual setup of the core unit constituting the *Coherence Monitor*.

Optional peripherals for extended modes of measurements, Fig. S3 (Madl et al. 2016):

**LED-Cascade**: The setup enables stimulation of samples via a Pulse-Width-Modulated constant current source of LED-cascades in four different wavelengths (narrow-band high-power LEDs with 395/620/515/460 nm); triple color R/G/B LEDs (12 x 3 x1 W) and mono color UV LEDs (12 x 1 W) - each wavelength can be used separately or in combinations.

**Strobe-light**: a continuous spectrum white light source that can be set to four different flashing rates (1/2/4/8 f/s).

**Thermoelectric unit**: Samples can be thermally stressed (either heated or cooled) via a tunable supply unit that powers two 90 W Peltier-Elements (PE-block).

**Sensor-Unit**: the dark-chamber is fitted with one rH and two T-sensors.

**Filterwheel (Atik-2)**: Emissions from samples can be measured in full spectral mode or by choosing specific emission windows in R/G/B or UV – filters and bandwidth can be swapped according to requirements.

**Interface (myDAQ)**: Both PMT-data acquisition as well as control of shutter and optional peripherals are achieved via two interface units (NI, 2016). Data logging is done via a standard desktop computer or laptop (not shown).


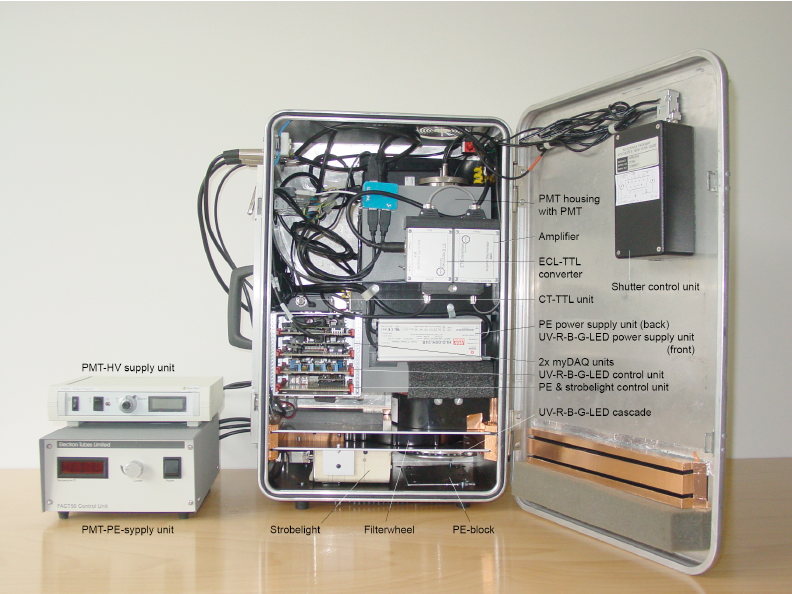


Fig. S3: extended peripherals to enable measurement of induced and delayed luminescence measurement mode of Coherence Monitor. It also features a thermoelectric block for abiotic stress induction of cell cultures (PE-block).

Reference (all web-sites access in Nov. 2016):

ET-Enterprises (2016a): Photon Counting System <http://www.et-enterprises.com/photon-detection-systems>

ET-Enterprises (2016b): [www.et-enterprises.com/files/file/PMT-accessories-brochures/accessories_1.pdf](http://www.et-enterprises.com/files/file/PMT-accessories-brochures/accessories_1.pdf)

NI (2016) myDAQ User Manual; www.ni.com/pdf/manuals/373060g.pdf

Madl P., Lehner B., Meyer P., Sereni P. (2016) Steuerung eines Detektors durch LabView® für Zellexpositionsstudien (Controlling detector components via LabView® for cell exposure studies). Begleitband zur 21^ten^ Tagung Virtuelle Instrumente in der Praxis (Proceedings of the 21^st^ Gathering of Virtual Instruments in Practical Use), Munich, FRG: 428-432.

<http://biophysics.sbg.ac.at/talk/CM-prototype.pdf>

Melles-Griot (2016) High Speed Shutters & Controllers [www.mellesgriot.com/media/b4c4f091-419e-49e5-b1a1-07c193912877/5Wr85g/PDFs/Shutter%20Brochure%202014_LR.pdf](http://www.mellesgriot.com/media/b4c4f091-419e-49e5-b1a1-07c193912877/5Wr85g/PDFs/Shutter Brochure 2014_LR.pdf)
